# Tox4 modulates cell fate reprogramming

**DOI:** 10.1242/jcs.232223

**Published:** 2019-10-15

**Authors:** Lotte Vanheer, Juan Song, Natalie De Geest, Adrian Janiszewski, Irene Talon, Caterina Provenzano, Taeho Oh, Joel Chappell, Vincent Pasque

**Affiliations:** KU Leuven - University of Leuven, Department of Development and Regeneration, Herestraat 49, B-3000 Leuven, Belgium

**Keywords:** Tox4, Induced pluripotent stem cells, Pluripotency, Reprogramming

## Abstract

Reprogramming to induced pluripotency induces the switch of somatic cell identity to induced pluripotent stem cells (iPSCs). However, the mediators and mechanisms of reprogramming remain largely unclear. To elucidate the mediators and mechanisms of reprogramming, we used a siRNA-mediated knockdown approach for selected candidate genes during the conversion of somatic cells into iPSCs. We identified *Tox4* as a novel factor that modulates cell fate through an assay that determined the efficiency of iPSC reprogramming. We found that *Tox4* is needed early in reprogramming to efficiently generate early reprogramming intermediates, irrespective of the reprogramming conditions used. *Tox4* enables proper exogenous reprogramming factor expression, and the closing and opening of putative somatic and pluripotency enhancers early during reprogramming, respectively. We show that the TOX4 protein assembles into a high molecular form. Moreover, *Tox4* is also required for the efficient conversion of fibroblasts towards the neuronal fate, suggesting a broader role of *Tox4* in modulating cell fate. Our study reveals *Tox4* as a novel transcriptional modulator of cell fate that mediates reprogramming from the somatic state to the pluripotent and neuronal fate.

This article has an associated First Person interview with the first author of the paper.

## Abstract (precis)

**Highlighted Article:** Discovery of *Tox4* as a mediator of reprogramming of mouse embryonic fibroblasts to the induced pluripotent and neuronal fate.

## INTRODUCTION

The discovery that somatic cells can be reprogrammed to a pluripotent state via somatic cell nuclear transfer or transcription factor (TF) expression approaches has revolutionized biology and regenerative medicine (Gurdon et al., 1958; Takahashi and Yamada, 2006). The overexpression of *Oct4*, *Klf4*, *Sox2* and *Myc* (collectively OKSM) reprograms somatic cells to become induced pluripotent stem cells (iPSCs), which are able to differentiate into all embryonic lineages including the germline (Wernig et al., 2007). iPSCs allow patient-specific disease modeling, drug screening and the derivation of functional cell types for regenerative medicine (Kim, 2015). iPSCs are entering clinical trials for multiple disorders including age-related macular degeneration (Mandai et al., 2017), Parkinson's disease (Barker et al., 2017) and diabetes (Sneddon et al., 2018). This reprogramming system also serves as a tool to broaden our understanding of how cell identity and cell fate transitions are regulated (Apostolou and Hochedlinger, 2013; Papp and Plath, 2013). However, somatic cells are resistant to reprogramming, which complicates mechanistic studies of reprogramming by lowering the efficiency of reprogramming (Hanna et al., 2009; Pasque et al., 2011).

Efforts to analyze factors involved in converting one type of somatic cell into another have revealed facilitators and barriers involved in the reprogramming process (Ebrahimi, 2015; Peñalosa-Ruiz et al., 2019). Screening approaches have identified pathways that act as barriers to reprogramming, such as the DNA damage response (Ocampo et al., 2016; Peñalosa-Ruiz et al., 2019), TGF-β signaling (Samavarchi-Tehrani et al., 2010), the chromatin modifier DOT1L (Onder et al., 2012), protein ubiquitylation (Buckley et al., 2012) and tri-methylation of histone H3 lysine 9 (H3K9me3) (Chen et al., 2013; Sridharan et al., 2013; Chronis et al., 2017), and factors that enhance reprogramming, such as proliferation (Ruiz et al., 2011; Son et al., 2013), the TF-encoding genes *Glis1*, *Cebpa* and *Esrrb* (Maekawa et al., 2011; Soufi et al., 2012; Brumbaugh et al., 2018) and small molecules, including ascorbic acid (AA) (Esteban et al., 2010). Technical advances, such as genome-wide screens, have enabled the comprehensive identification of pathways and factors that impede reprogramming, for example, clathrin-mediated endocytosis (Qin et al., 2014), *Nfe2* (Yang et al., 2014), *Chaf1a* (Cheloufi et al., 2015), sumoylation (Borkent et al., 2016) and polyadenylation (Brumbaugh et al., 2018). However, despite these advances, functional validation of targets and a mechanistic understanding of cell state transitions during reprogramming remains incomplete. Furthermore, while screens performed in pluripotent stem cells have identified regulators required to maintain pluripotency (Kaji et al., 2006; Pereira et al., 2006; Betschinger et al., 2013; Leeb et al., 2014; Ding et al., 2015; Li et al., 2018; Yilmaz et al., 2018), it often remains unclear whether the same factors also play a role in induction of pluripotency during cell fate reprogramming, independent of their function in maintaining pluripotency.

Work by several laboratories has indicated that reprogramming is a stepwise process with many cellular intermediates (Stadtfeld et al., 2008; Buganim et al., 2012; Polo et al., 2012; Hussein et al., 2014; Pasque et al., 2014; Guo et al., 2019; Schiebinger et al., 2019). During reprogramming, cells initially undergo a mesenchymal-to-epithelial transition (Li et al., 2010; Samavarchi-Tehrani et al., 2010). This is followed by upregulation of the polycomb repressive complex 2 (PRC2) protein enhancer of zeste 2 (EZH2) during intermediate reprogramming stages (Pasque et al., 2014), then the activation of early pluripotency genes, such as *Nanog* (Stadtfeld et al., 2008; Buganim et al., 2012; Guo et al., 2019). Completion of induced pluripotency takes place late in reprogramming and includes hierarchical reactivation of pluripotency genes, including *Dppa4*, the activation of which takes place in true iPSCs (Buganim et al., 2012; Golipour et al., 2012; Polo et al., 2012; Pasque et al., 2014). In addition, dynamic chromatin remodeling assists cis-regulatory control of gene expression and associated changes in target-binding sites of TFs and thereby further modulates reprogramming (Chronis et al., 2017; Zviran et al., 2019). Because cells undergo many state transitions during reprogramming (Pasque et al., 2014; Guo et al., 2019; Schiebinger et al., 2019), it is imperative to identify and examine the role of selected reprogramming barriers and facilitators in different stages of reprogramming. Recent studies aiming to account for the presence of distinct reprogramming intermediates have revealed additional regulators following functional interference (Toh et al., 2016; Schwarz et al., 2018; Peñalosa-Ruiz et al., 2019). Nevertheless, most mechanistic reprogramming studies have examined only one reprogramming stage, and the heterogeneity due to the presence of many reprogramming stages may have obscured mechanistic studies. Single-cell studies have resolved cellular heterogeneity, but mechanisms remain enigmatic (Guo et al., 2019; Schiebinger et al., 2019; Tran et al., 2019). Thus, facilitators and barriers to specific cell state transitions during reprogramming remain incompletely identified and understood. Moreover, the use of different reprogramming systems between and within laboratories can lead to distinct responses upon modulation of candidate facilitator or barrier to reprogramming (Chantzoura et al., 2015). Finally, the culture conditions used for reprogramming, in particular AA, may influence reprogramming outcomes (Esteban et al., 2010). An approach in which reprogramming is analyzed in different reprogramming stages would increase our ability to perform mechanistic studies.

Here, we used small interfering RNA (siRNA)-mediated knockdown of candidate genes during the induction of iPSCs from mouse embryonic fibroblasts in order to identify novel modulators of reprogramming to induced pluripotency. We uncovered *Tox4*, a high mobility group (HMG) box transcriptional regulator, as a novel factor needed for efficient reprogramming of fibroblasts towards both the pluripotent and neuronal fate. By systematically examining specific reprogramming intermediates in different reprogramming conditions, we found that *Tox4* is involved early during reprogramming, before pluripotency is reached, to ensure proper exogenous OKSM expression and changes in chromatin accessibility.

## RESULTS

### Candidate gene knockdown identifies *Tox4* as a modulator of cell fate reprogramming

**Fig. 1.**
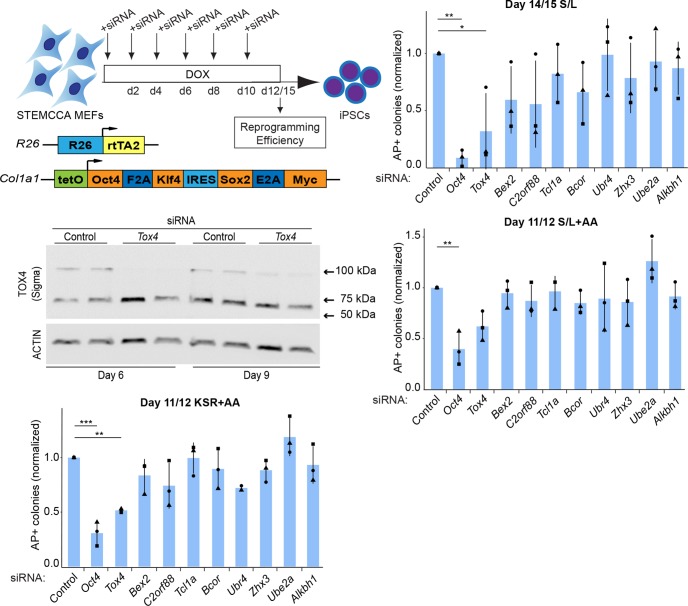
**siRNA screen for modulators of reprogramming to iPSCs identifies *Tox4* as a novel modulator of reprogramming.** (A) Schematic of targeted siRNA approach for modulators of reprogramming to iPSCs. Target genes were targeted every other day by siRNA transfection of STEMCCA MEFs induced to reprogram. ‘STEMCCA’ MEFs allow for a DOX-inducible expression of *Oct4*, *Sox2*, *Klf4* and *Myc* resulting in the generation of iPSCs. (B) The number of AP+ colonies at D14 or 15 of reprogramming in S/L with no AA. Colony counts were normalized to colony counts in control conditions. Results are shown as the mean±s.d. (*n*=3 with two biological replicates in total). *P<0.05; **P<0.01 (one-way ANOVA with Dunnett's multiple comparisons test compared to control). (C) Western blot analysis for TOX4 (Sigma antibody) and actin after 6 days and 9 days of STEMCCA MEFs reprogramming and transfection of *Tox4* or control siRNAs every other day. (D) The number of AP+ colonies at D11 or 12 of reprogramming in S/L+AA. Colony counts were normalized to colony counts in control conditions. Results are shown as the mean±s.d. (*n*=3 with two biological replicates in total). **P<0.01 (one-way ANOVA with Dunnett's multiple comparisons test compared to control). (E) The number of AP+ colonies at D11 or 12 of reprogramming in KSR+AA. Colony counts were normalized to colony counts in control conditions. Results are shown as the mean±s.d. (*n*=3 with two biological replicates in total). **P<0.01, ***P<0.001 (one-way ANOVA with Dunnett's multiple comparisons test compared to control). Squares, triangles and circles represent one independent experiment each.

To define factors that modulate fibroblast reprogramming to iPSCs, we knocked down candidate genes by RNA interference (RNAi) in ‘STEMCCA’ mouse embryonic fibroblasts (MEFs), derived from mice heterozygous for *Col1a1-tetO-OKSM* and heterozygous for *Rosa26-M2rtTA* (Fig. 1A) (Sridharan et al., 2013). This system enables doxycycline (DOX)-inducible expression of OKSM from a polycistronic cassette and results in the generation of iPSCs with all known molecular and functional properties of naive pluripotency (Carey et al., 2010; Stadtfeld et al., 2010; Sridharan et al., 2013).

To identify modulators of reprogramming, we selected ten candidate genes for targeting with siRNAs. *Oct4* was chosen as a control because it is required for reprogramming (Takahashi and Yamanaka, 2006). *Tox4* was chosen because its role in pluripotency induction in unknown and it has been implicated in maintenance of pluripotency (Ding et al., 2015). *Bex2*, *C2orf88* and *Tcl1a* were chosen based on gene expression because they are amongst the most upregulated genes in embryonic stem cells (ESCs) compared with MEFs (Chronis et al., 2017). *Ube2a*, *Ubr4* and *Bcor* were chosen because they have been implicated as reprogramming barriers, but their precise role remains unclear (Cheloufi et al., 2015). *Alkbh1* was picked because it has been reported as an adenine demethylase that might regulate cell fate reprogramming (Xiao et al., 2018). *Zhx3* was selected because it is a homeobox TF expressed in blastocysts but its potential role in reprogramming has not been investigated (Guo et al., 2017).

Reprogramming was carried out in ESC medium with 15% fetal bovine serum (FBS) and leukemia inhibitory factor (LIF) (denoted throughout as S/L). siRNAs were transfected every other day throughout the reprogramming process. At day 14 or 15, reprogramming efficiency was assessed using alkaline phosphatase (AP) staining (Fig. 1A). We observed a decrease in the number of AP positive (+) colonies for the *Oct4* control (Fig. 1B). As expected, we also observed a decrease with previously reported regulators, such as *Bex2* (Schwarz et al., 2018), *C2orf88*, *Tcl1a*, *Bcor* and *Zhx3*, but the effect was not significant (Fig. 1B). Surprisingly, *Ube2a* depletion did not increase reprogramming efficiency, in contrast with what was found in a previous study (Cheloufi et al., 2015). *Tox4* depletion significantly decreased the number of AP+ colonies. *Tox4* is considered to be involved in maintenance of pluripotency (Ding et al., 2015), but had not previously been shown to influence induction of pluripotency. We have therefore identified *Tox4* as a potential modulator of reprogramming to iPSCs, and focus on this factor for the remainder of the study.

We confirmed that *Tox4* transcript and protein levels were downregulated in *Tox4* siRNA-treated cells (Fig. 1C; Fig. S1A). Surprisingly, despite a predicted molecular mass of TOX4 protein of 66 kDa, western blot analysis under denaturing conditions using two independent antibodies revealed the presence of a 100 kDa band, which was consistently decreased specifically upon *Tox4* siRNA transfection (Fig. S1B,C). To confirm the specificity of this 100 kDa band, we tagged the N- or C-terminus of TOX4 with human influenza hemagglutinin (HA) tags in mouse ESCs followed by western blot with anti-HA antibodies. Western blot analysis against HA revealed a single 100 kDa band in ESCs expressing exogenous HA-tagged *Tox4*, suggesting that *Tox4* has a higher than predicted molecular weight (Fig. S1D). Altogether, these data confirm the efficient depletion of TOX4 protein in our knockdown experiments.

Culture conditions modulate reprogramming, hence, it is important to test whether the effects of functional studies are culture media-specific or globally applicable (Esteban et al., 2010; Liu et al., 2014a,b). Therefore, we conducted a secondary siRNA screen in AA and knockout serum replacement (KSR) conditions, which both strongly enhance reprogramming efficiency (Esteban et al., 2010; Liu et al., 2014a,b). When AA or KSR was used during reprogramming, there was a rescue, or partial rescue, of the effect of siRNA knockdown for most targeted genes (Fig. 1D,E). *Ube2a* knockdown seemed to increase reprogramming efficiency in the presence of AA (Fig. 1D), in agreement with *Ube2a* acting as a barrier to reprogramming (Cheloufi et al., 2015), but not in the absence of AA (Fig. 1B), suggesting an AA-dependent effect. In contrast, *Tox4* knockdown impeded efficient reprogramming, independently of the reprogramming conditions used, with effects nearly similar to those of *Oct4* knockdown (Fig. 1D,E). Consistent with these findings, the number of DPPA4+ colonies, a stringent marker of late reprogramming stages, was decreased at day 12 of reprogramming upon *Tox4* suppression, albeit non significantly (Fig. S1E,F) (Pasque et al., 2014). These results imply that *Tox4* suppression impedes efficient reprogramming and the formation of late reprogramming intermediates in low- and high-efficiency reprogramming conditions.

### *Tox4* depletion prevents the formation of early reprogramming intermediates

**Fig. 2.**
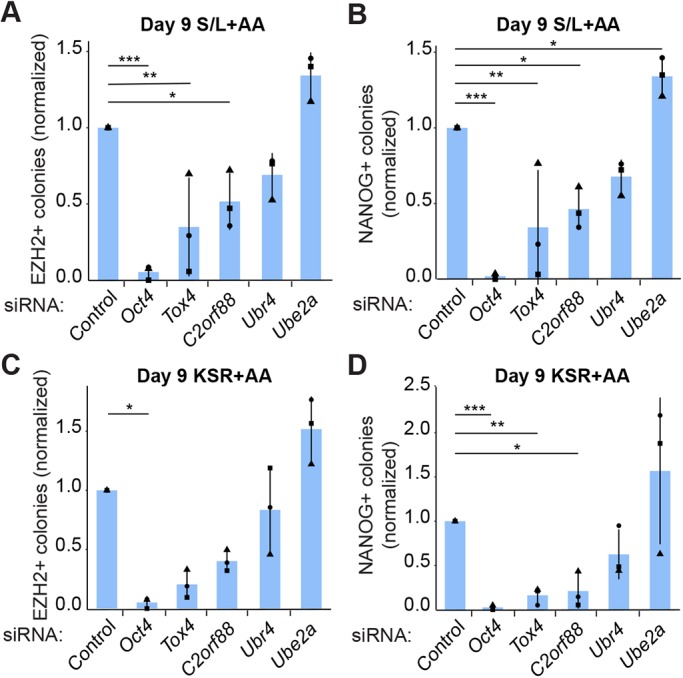
***Tox4* suppression impedes intermediate reprogramming stages.** (A–D) The indicated genes were targeted every other day by siRNA transfection of STEMCCA MEFs induced to reprogram. (A) The number of EZH2+ colonies at D9 of reprogramming in S/L+AA. Colony counts were normalized to colony counts in control conditions. Results are shown as the mean±s.d. (*n*=3 with two biological replicates in total). *P<0.05, **P<0.01, ***P<0.001 (one-way ANOVA with Dunnett's multiple comparisons test compared to control). (B) The number of NANOG+ colonies at D9 of reprogramming in S/L+AA. Colony counts were normalized to colony counts in control conditions. Results are shown as the normalized mean±s.d. of two independent experiments. Results are shown as the mean±s.d. (*n*=3 with biological duplicates in total). *P<0.05, **P<0.01, ***P<0.001 (one-way ANOVA with Dunnett's multiple comparisons test compared to control). (C) Same as Fig. 2A for KSR+AA. (D) Same as Fig. 2B for KSR+AA. Squares, triangles and circles represent one independent experiment each.

Next, to further refine reprogramming kinetics upon *Tox4* suppression, we set out to define whether early reprogramming stages were affected. Therefore, we analyzed the formation of EZH2+ and NANOG+ colonies, which are indicative of early and intermediate reprogramming intermediates, respectively (Pasque et al., 2014). Following *Tox4* knockdown during reprogramming, the number of EZH2+ and NANOG+ colonies was significantly reduced (Fig. 2A,B). This effect seemed more pronounced in KSR+AA (Fig. 2C,D) than in S/L+AA (Fig. 2A,B) conditions, in agreement with the reduced formation of late reprogramming intermediates under the same conditions (Fig. 1D,E). Thus, in addition to its role in maintaining pluripotency (Ding et al., 2015), *Tox4* is involved in the induction of reprogramming towards pluripotency.

Based on these findings, we explored the ability of *Tox4* to promote reprogramming. We used pre-iPSCs, which are incompletely reprogrammed clonal cell lines obtained after expression of OKSM from individual exogenous viruses in the presence of serum, which can then be fully reprogrammed toward naive pluripotency upon dual inhibition of GSK3B and ERK1/2 in the presence of LIF (denoted 2i/L) (Silva et al., 2008; Tonge et al., 2014). We overexpressed *Tox4* in pre-iPSCs and switched the cells to 2i/L+KSR conditions for 9 days and then undertook a NANOG+ colony count. *Tox4* overexpression was validated at the transcript and protein levels (Fig. S2A,B). No difference in reprogramming efficiency was observed as a result of overexpressing TOX4 (Fig. S2C). These results suggest that *Tox4* enables reprogramming, but its overexpression does not promote the acquisition of naive pluripotency starting from pre-iPSCs.

### Somatic *Tox4* enables the conversion of somatic cells into iPSCs

**Fig. 3.**
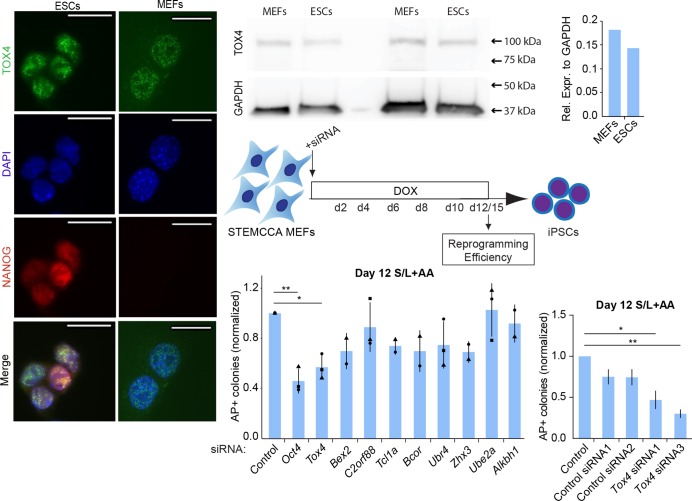
***Tox4* suppression impedes intermediate reprogramming stages.** (A) Immunofluorescence analysis for TOX4/NANOG in ESCs grown in S/L and MEFs, showing expression and nuclear localization in both cell types. Representative images of all lines examined for TOX4 (green), NANOG (red) and DAPI (blue, nuclei counterstaining) are shown. Scale bars: 20 µm. (B) Western blot for TOX4 (Sigma) and GAPDH in MEFs and ESCs. (C) Quantification of TOX4 western blot analysis using GAPDH as a loading control. Results are shown as the mean of technical duplicates (*n*=1). (D) Schematic of siRNA-mediated somatic *Tox4* knockdown at the start of reprogramming to iPSCs. Indicated genes were targeted at D0 by siRNA transfection of STEMCCA MEFs after subsequent DOX induction of reprogramming. (E) The number of AP+ colonies at D12 of reprogramming in S/L+AA. Results for control, *Oct4*, *Tox4*, *C2Orf88*, *Ubr4* and *Ube2a* siRNA are shown as mean±s.d. (*n*=2 or 3 with biological duplicates in total). Results for *Bex2*, *Tcl1a*, *Bcor*, *Zhx3* and *Alkbh1* siRNA are shown as the mean±s.d. (*n*=2 with two biological replicates in total). *P<0.05, **P<0.01 (one-way ANOVA with Dunnett's multiple comparisons test compared to control). (F) The number of AP+ colonies at D12 of reprogramming in S/L+AA. Counts were normalized to counts in control conditions. Results are shown as the normalized mean±s.d. (*n*=1 with biological duplicates in total). Squares, triangles and circles represent one independent experiment each.

Next, we asked whether somatic TOX4 mediates reprogramming towards iPSCs. Immunofluorescence analysis revealed nuclear TOX4 protein in both MEFs and ESCs, confirming somatic expression of TOX4 (Fig. 3A). Somatic expression of TOX4 is consistent with reports in other somatic cell types (Nagase et al., 1998). Expression of TOX4 protein in ESCs corroborates a study on *Tox4* in pluripotency maintenance (Ding et al., 2015). Western blot analysis revealed similar levels of TOX4 protein in MEFs and ESCs (Fig. 3B,C). To determine whether TOX4 mediates early reprogramming, we performed a single round of siRNA transfection in STEMCCA MEFs, followed by induction of reprogramming. Reprogramming efficiency was measured using AP staining at day 15 (Fig. 3D). Lower reprogramming efficiency correlated with *Tox4* depletion at the start of reprogramming (Fig. 3E). Thus, somatic TOX4 is needed for efficient reprogramming to iPSCs. To exclude the possibility that previous observations were influenced by off-target effects of pooled *Tox4* siRNAs, we knocked down somatic *Tox4* using a single round of individual *Tox4* siRNA transfection at the start of reprogramming. *Tox4* suppression using single siRNAs lowered *Tox4* transcript level and decreased the formation of early and intermediate reprogramming markers (Fig. 3F; Fig. S3) consistent with previous findings (Fig. 2). Thus, suppression of *Tox4* at an early stage is sufficient to reduce efficient reprogramming to iPSCs.

### *Tox4* suppression prolongs the expression of selected somatic genes early during reprogramming

**Fig. 4.**
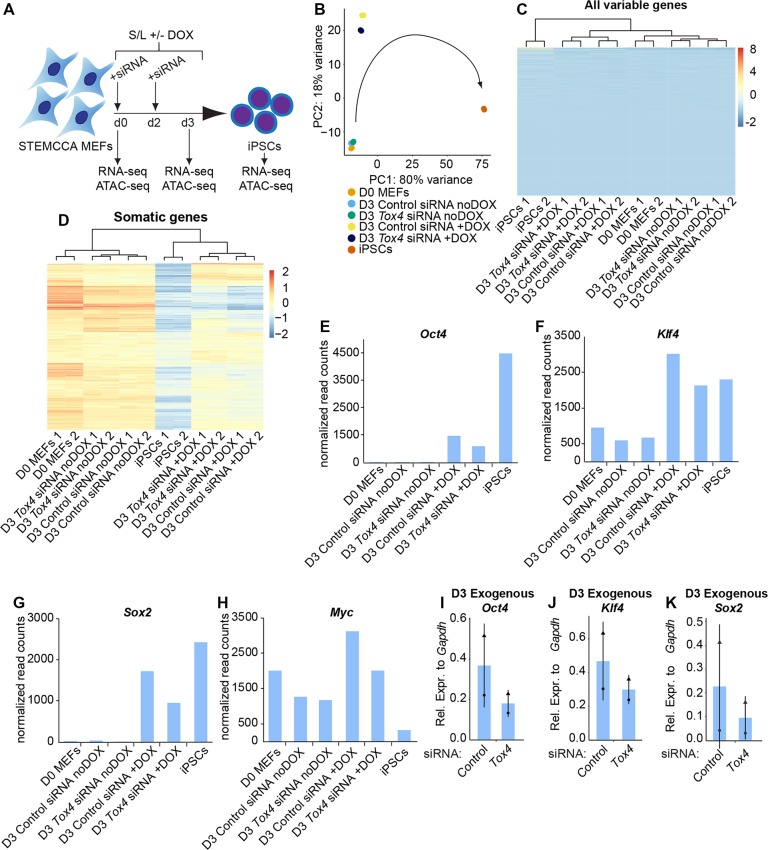
***Tox4* suppression prolongs the expression of selected somatic genes early during reprogramming.** (A) Scheme of *Tox4* knockdown during reprogramming to iPSC in S/L with and without DOX. Samples for RNA-seq and ATAC-seq were collected at D0 and D3 of reprogramming. In parallel, iPSCs without siRNA treatment were collected after 12 days of DOX induction and were included as a control. (B) PCA of the 500 most variable genes across all samples. Each point represents a single sample and is labeled according to sample name. Data were plotted along the first and second principal components. The arrow indicates the trajectory of the reprogramming time course. (C) Unsupervised hierarchical clustering of all variable genes across all samples. Normalized gene expression was plotted on a high-to-low scale (red–blue). (D) Unsupervised hierarchical clustering of somatic genes across all samples suggesting that the expression of a subset of somatic genes is elevated in *Tox4* siRNA-treated cells. Somatic genes were defined as the top 500 genes that were significantly (*P*<0.05) more highly expressed in D0 MEFs compared to iPSCs in this dataset. Normalized gene expression was plotted on a high-to-low scale (red–blue). (E–H) Normalized read counts of *Oct4* (E), *Klf4* (F), *Sox2* (G) and *Myc* (H) in early reprogramming to iPSCs. Results are shown as the mean of technical duplicates (*n*=1). (I–K) Exogenous *Oct4* (I), *Klf4* (J) and *Sox2* (K) transcript level after 3 days of STEMCCA MEFs reprogramming and transfection of *Tox4* or control siRNAs every 2 days. Results are shown as the normalized mean±s.d. relative to the expression of *Gapdh* (arbitrary units) (*n*=2 with biological duplicates in total). Squares, triangles and circles represent one independent experiment each.

To gain insight into how *Tox4* suppression affects early reprogramming to induced pluripotency at the transcriptional level, we performed duplicate RNA sequencing (RNA-seq) of STEMCCA MEFs before induction of reprogramming [Day 0 (D0)], and three days after induction of reprogramming in the presence of either *Tox4* or control siRNAs (D3 +DOX), as well as D3 controls without DOX (D3 noDOX) (Fig. 4A). Principal component analysis (PCA) and unsupervised clustering of all variable genes revealed that in the absence of DOX, fibroblasts maintained a fibroblast-like transcriptome in the presence of *Tox4* knockdown (Fig. 4B,C). Upon induction of reprogramming, *Tox4* knockdown did not result in global changes in gene expression compared with control cells. We confirmed that *Tox4* transcript levels were downregulated in *Tox4* siRNA-treated cells based on RNA-seq data (Fig. S4A).

Previous studies have shown that fibroblasts downregulate the somatic program early during reprogramming (Stadtfeld et al., 2008; Polo et al., 2012). Therefore, we assessed whether *Tox4* suppression prolongs the expression of the somatic program, and thereby potentially hinders efficient reprogramming to induced pluripotency. We performed unsupervised clustering based on somatic gene expression, defined as genes which were significantly more expressed in MEFs compared to iPSCs (Table S1). Indeed, we observed that *Tox4* depletion resulted in a delay in the downregulation of a subset of somatic genes compared to control conditions (Fig. 4D, Fig. S4B–G). Surprisingly, even in the absence of DOX, somatic gene expression was increased in the *Tox4* knockdown condition compared to control conditions, with the exception of *Crim1*, indicating that *Tox4* influences gene expression in the absence of induction of reprogramming. Altogether, these findings show that *Tox4* suppression prolongs the expression of a subset of somatic genes.

Successful reprogramming has been attributed to high levels of ectopic OKSM expression (Tiemann et al., 2011). In addition, *Tox4* has been shown to interact with the polymerase associated factor 1 complex (PAF1C), which is involved in transcription initiation and elongation (Ding et al., 2015). This raises the question of whether *Tox4* suppression alters ectopic OKSM expression. Therefore, we analyzed OKSM transcript levels at early reprogramming time points. Under *Tox4* knockdown conditions, we observed that *Tox4* suppression correlated with lower exogenous OKSM expression, which we confirmed by quantitative real-time PCR (RT-qPCR; Fig. 4E–K). In summary, this data implies that *Tox4* suppression disturbs exogenous OKSM induction and therefore might hamper efficient reprogramming to induced pluripotency.

To exclude the possibility that previous observations are unique to DOX inducible systems, we induced the reprogramming of MEFs by infection with retroviruses encoding for *Oct4*, *Sox2* and *Klf4*. After initial retroviral infection, *Tox4* was knocked down every other day. After 17 days, reprogramming efficiency was assessed by AP staining (Fig. S4H). *Tox4* suppression by siRNA lowered *Tox4* transcript level and decreased the number of AP+ colonies (Fig. S4I,J). Therefore, *Tox4* knockdown affects reprogramming even in DOX-independent reprogramming systems.

High proliferation rates have been associated with successful reprogramming (Ruiz et al., 2011; Son et al., 2013). Given the reported interaction of TOX4 with known cell cycle modulators such as PAF1C and protein phosphatase 1 (PP1) (Koch et al., 1999; Neganova and Lako, 2008; Ding et al., 2015), we hypothesized that proliferation rates may be altered upon *Tox4* knockdown. Indeed, the transcript levels of several cyclins such as *Cdk1*, *Cdk2*, *Ccna1*, *Ccne1* and *Ccne2* and the proliferation marker *Mki67* (Gérard and Goldbeter, 2012; Sun et al., 2017) were decreased upon *Tox4* knockdown compared to control conditions, indicative of potentially altered cell cycle progression and decreased proliferation (Fig. S5A–F). To assess proliferation upon *Tox4* knockdown, we performed a single round of siRNA transfection in STEMCCA MEFs, followed by induction of reprogramming and Carboxyfluorescein succinimidyl ester (CFSE) staining to assess proliferation rate by flow cytometry at D4 (Fig. S5G). The CFSE staining showed that *Tox4* siRNA-treated cells proliferated at a slower rate compared to control conditions (Fig. S5H,I). Additional cell cycle analysis by 5-ethynyl-2′-deoxyuridine (EdU) and 4′,6-diamidino-2-phenylindole (DAPI) flow cytometry revealed no difference in cell cycle distribution and a lower number of dividing cells for *Tox4* siRNA-treated cells compared to the control, consistent with the CFSE staining (Fig. S5J–M). Gene ontology analysis of significantly downregulated genes in *Tox4* siRNA-treated cells revealed terms associated with ‘G1/S transition of mitotic cell cycle’, ‘G_2_/M DNA replication checkpoint’ and ‘DNA replication initiation’, consistent with gene expression changes (Fig. S5A–F, Tables S2–S5). Altogether, these findings show that *Tox4* suppression slows down proliferation, potentially affecting reprogramming efficiency.

### *Tox4* suppression delays the closing of somatic and opening of pluripotency chromatin regions

**Fig. 5.**
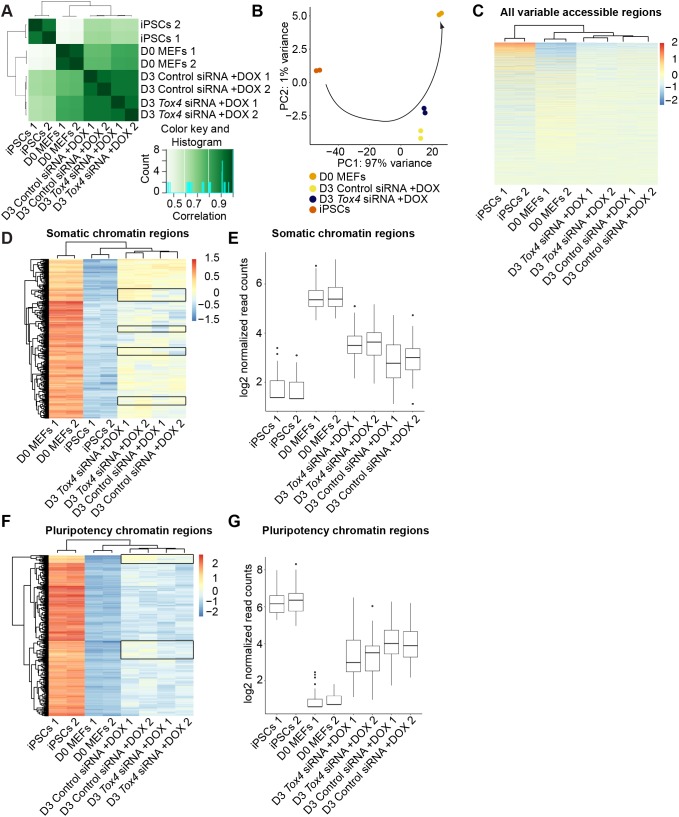
***Tox4* knockdown modulates chromatin accessibility early during reprogramming.** (A) ATAC-seq sample-to-sample distance heatmap showing the Euclidean distance between samples showing that *Tox4* siRNA-treated cells are more similar to D0 MEFs compared to the control condition. (B) PCA of the all variable accessible chromatin regions across all samples. Each point represents a single sample and is labeled according to sample name. Data were plotted along the first and second principal components. The arrow indicates the trajectory of the reprogramming time course. (C) Unsupervised hierarchical clustering of all variable accessible chromatin regions for D0 and D3 MEFs, and iPSCs. Normalized read counts was plotted on a high-to-low scale (red–blue). (D) Unsupervised hierarchical clustering of somatic accessible chromatin regions for D0 and D3 MEFs, and iPSCs implying that Tox4 knockdown delays the closing of a subset of somatic accessible chromatin regions. Somatic accessible regions were defined as the top 500 regions that were significantly (*P*<0.05) more highly expressed in D0 MEFs compared to iPSCs in this dataset. Normalized read counts was plotted on a high-to-low scale (red–blue). Boxes indicate the subset of somatic accessible regions used in E. (E) Box plot of chromatin accessibility, indicated by log2-transformed normalized read counts, of the subset of putative somatic enhancers that were more accessible in Tox4 siRNA-treated cells compared to control conditions (*n*=1). (F) Unsupervised hierarchical clustering of pluripotency accessible regions for D0 and D3 MEFs, and iPSCs. Pluripotency accessible regions were defined as the top 500 regions significantly more open in iPSCs compared to MEFs when sorting based on log2 fold change (*P*<0.05) in this dataset. Normalized gene expression was plotted on a high-to-low scale (red–blue). Boxes indicates the subset of pluripotency accessible regions used in G. (G) Box plot of chromatin accessibility, indicated by log2-transformed normalized read counts, of the subset of putative pluripotency enhancers that were less accessible in Tox4 siRNA-treated cells compared to control conditions (*n*=1). In E and G, boxes correspond to the 25th and 75th quartiles, horizontal lines to the median, and whiskers extend to 1.5 times the interquartile range. Dots denote outliers.

Cis-regulatory control of gene expression is achieved by TF binding to target DNA sequences (Venkatesh and Workman, 2015). Such genomic regions often possess accessible chromatin (Slattery et al., 2014). To determine how *Tox4* suppression affects chromatin remodeling at the early stages of reprogramming to induced pluripotency, we used the assay for transposase accessible chromatin sequencing (ATAC-seq) (Fig. 4A). At D3 of reprogramming, the open chromatin landscape resembled the somatic state more than the iPSC state (Fig. 5A). This corresponded with RNA-seq results where D3 reprogramming cultures were transcriptionally more similar to MEFs than iPSCs. These results are consistent with changes in chromatin accessibility taking place before global transcriptome changes. As judged by PCA and unsupervised clustering, *Tox4* suppression did not result in global changes in chromatin accessibility (Fig. 5B,C).

We then analyzed chromatin accessibility specifically at MEF and ESC open chromatin regions. We performed unsupervised clustering based on somatic accessible regions, defined as regions that were significantly more open in MEFs compared to iPSCs (Fig. 5D; Table S6). Most chromatin regions behaved similarly between control and *Tox4* knockdown conditions. However, unsupervised clustering of somatic accessible regions revealed that *Tox4* depletion resulted in more accessible chromatin in a subset of somatic regions compared to the control siRNA condition (Fig. 5E, Table S7). Altogether, these findings imply that *Tox4* suppression delays the closing of a subset of somatic accessible chromatin regions, potentially delaying efficient reprogramming to induced pluripotency.

During later stages of reprogramming, the endogenous pluripotency network needs to be reactivated in order to acquire a stable pluripotent stem cell state that is independent of exogenous OKSM expression (Polo et al., 2012; Chronis et al., 2017). Therefore, we asked whether there is a delay in the opening of pluripotency accessible chromatin after *Tox4* depletion. We performed unsupervised clustering based on pluripotency-specific open regions, defined as regions which were significantly more open in iPSCs compared to MEFs (Fig. 5F; Table S8). Indeed, we observed that *Tox4* depletion resulted in less accessible chromatin at a subset of pluripotency regions compared to control conditions (Fig. 5G; Table S9). Pluripotency accessible chromatin that opened with a delay was associated with genes such as *Cdh1*, *Cdh2* and *Chd1*, with known functions in reprogramming and pluripotency (Table S10) (Gaspar-Maia et al., 2009; Takehara et al., 2015; An et al., 2017). In summary, *Tox4* depletion disturbs the opening of a subset of pluripotency-related regions, which may help to explain less-efficient reprogramming to induced pluripotency.

### *Tox4* suppression limits transdifferentiation to the neuronal fate

**Fig. 6.**
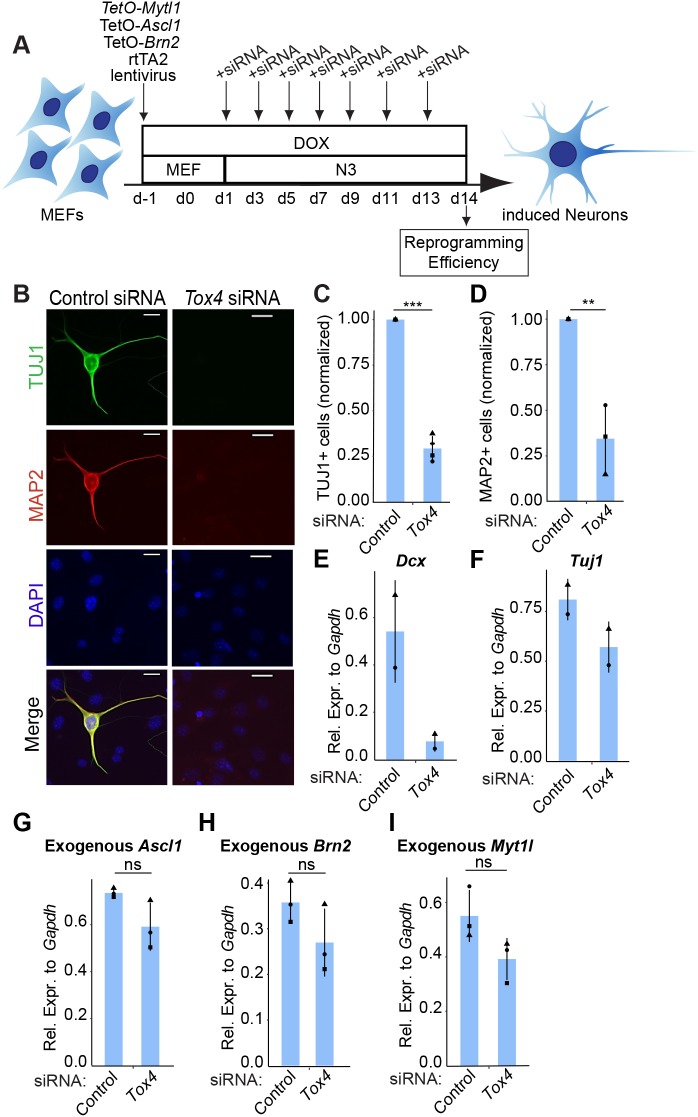
***Tox4* depletion hinders the efficient transdifferentiation of fibroblasts to the neuronal fate.** (A) Scheme of siRNA-mediated *Tox4* knockdown throughout the reprogramming of fibroblasts to induced neurons. (B) Immunofluorescence analysis for TUJ1 andMAP2 at D14 of transdifferentiation. Induced neurons were defined as TUJ1+ cells if cells had processes at least three times longer than the cell body. Representative images of all lines examined for TUJ1 (green), MAP2 (red) and DAPI (blue, nuclei counterstaining) are shown. Scale bars: 20 µm. (C) The number of TUJ1+ colonies at D14 of transdifferentiation. Counts were normalized to counts in control conditions. Results are shown as the normalized mean±s.d. (*n*=4 with 1 biological replicate in total). ***P<0.001 (two-tailed unpaired *t*-test). (D) Same as Fig. 6C for MAP2 (*n*=3 with 1 biological replicate in total). **P<0.01 (two-tailed unpaired *t*-test). (E,F) *Dcx* (E) and *Tuj1* (F) transcript level after 14 days of transdifferentiation and transfection of *Tox4* or control siRNAs every 2 days. Results are shown as the normalized mean±s.d. relative to the expression of *Gapdh* (arbitrary units) (*n*=2 with 1 biological replicate in total). (G–I) Exogenous *Ascl1* (G), *Brn2* (H) and *Myt1 l* (I) transcript level after 4 days of transdifferentiation and transfection of *Tox4* or control siRNAs every 2 days. Results are shown as the normalized mean±s.d. relative to the expression of *Gapdh* (arbitrary units) (*n*=3 with 1 biological replicate in total). ns, not significant (one-way ANOVA with Dunnett's multiple comparisons test compared to control). Squares, triangles, crosses and circles represent one independent experiment each.

We next investigated whether *Tox4* is also needed for alternative cell fate transitions that do not involve a pluripotent state. We reprogrammed wild-type (WT) MEFs into induced neurons (iNs) by ectopically expressing three neuronal-related TFs: *Ascl1*, *Brn2* and *Myt1l* (Vierbuchen et al., 2010). Transdifferentiation was initiated upon DOX addition concomitant with *Tox4* knockdown by siRNA transfection every other day (Fig. 6A). After 14 days, the formation of iNs, defined as TUJ1+ (recognizing TUBB3) and MAP2+ cells, was assessed by performing immunofluorescence microscopy to determine direct reprogramming efficiency (Vierbuchen et al., 2010). We confirmed that cell cultures were free of TUJ1+ and MAP2+ neurons before transdifferentiation was initiated, consistent with previous findings (Fig. S6A) (Vierbuchen et al., 2010). We confirmed that *Tox4* transcript levels were downregulated in *Tox4* siRNA-treated cells (Fig. S6B,C). *Tox4* knockdown throughout the reprogramming led to a significant decreased formation of TUJ1+ and MAP2+ iNs (Fig. 6B–D; Fig. S6D). As shown by RT-qPCR, the expression of neuronal markers *Dcx* and *Tuj1* tends to decrease upon *Tox4* knockdown, consistent with Fig. 6B–D (Fig. 6E,F). We also observed a trend towards decreased exogenous *Ascl1*, *Brn2* and *Myt1l* expression upon *Tox4* knockdown, albeit not significantly (Fig. 6G–I). Altogether, these results show that *Tox4* is not only needed for the efficient reprogramming of fibroblasts to iPSCs, but also for direct reprogramming into iNs.

## DISCUSSION

Reprogramming to iPSCs enables patient-specific disease modeling, regenerative medicine approaches, and broadens our understanding of the regulatory control of cell states and transitions. However, inefficiency, heterogeneity and multiple cell identity transitions complicate the elucidation of the mechanisms behind reprogramming. Despite several advances and extensive research, the mechanisms surrounding reprogramming remain unclear, in particular regarding cell state transitions. Here, we report a role of *Tox4* in cell fate reprogramming as shown by performing an siRNA-mediated knockdown of candidate genes using reprogramming to iPSCs as an experimental system. Analyses of early reprogramming intermediates as well as *Tox4* knockdown in the somatic cell state suggests a role for *Tox4* in early cellular reprogramming. Interestingly, a recent study reported that *Tox4* is needed to maintain pluripotency in ESCs and in epiblast stem cells (Ding et al., 2015). Thus, *Tox4* is required not only for pluripotency maintenance, but also for its establishment. In addition, we report a role for *Tox4* in the efficient transdifferentiation of fibroblasts towards a neuronal fate, implying a broader role of *Tox4* in modulating cell fate independently of whether cells pass through a self-renewing pluripotent stem cell state.

Mechanistically, *Tox4* seems to mediate ectopic OKSM expression, which in turn is essential for efficient reprogramming to induced pluripotency (Tiemann et al., 2011). Whether ectopic *Oct4*, *Sox2* and *Klf4* expression is also reduced in the retroviral experiment in the absence of DOX remains to be defined. Indeed, several studies have shown that large-scale chromatin changes, which will ultimately lead to the establishment of ESC-like chromatin, are mediated by ectopic OKSM expression levels throughout reprogramming (Hussein et al., 2014; Tonge et al., 2014; Knaupp et al., 2017). More specifically, OSK binds to active somatic enhancers early in reprogramming in order to induce the genome-wide inactivation of the somatic gene program (Polo et al., 2012; Chronis et al., 2017). Indeed, lower OKSM expression upon *Tox4* knockdown leads to a delay in the closing of a subset of somatic chromatin regions which would cause a delay in the inactivation of the somatic program. In addition, OSK has also been shown to engage pluripotency enhancers early in reprogramming in a stepwise manner (Chronis et al., 2017). This supports our observation that lower OKSM expression upon *Tox4* knockdown disturbs the proper opening of pluripotency accessible regions.

In this work, we defined the effect of *Tox4* expression using pre-iPSCs. However, the effect of *Tox4* overexpression on reprogramming of MEFs into iPSCs or iNs remains to be further investigated. Additionally, it would be interesting to assess the effect of *Tox4* overexpression and knockdown in alternative cell fate conversion systems, including the transdifferentiation of MEFs to trophoblast stem cells (Kubaczka et al., 2015).

At the molecular level, our results support the presence of TOX4 as a high molecular mass protein. This finding is consistent with reports of other HMG proteins possessing a high mobility box that engages in protein–protein interactions and binding to distorted DNA (O'Flaherty and Kaye, 2003), consistent with the formation of stable protein complexes. The reported interaction between TOX4 and PP1, a known regulator of transcription, chromatin regulation and cell cycle regulation (Lee et al., 2010; Ding et al., 2015) could explain the globally altered cell cycle progression. The latter has been described as rate-limiting during reprogramming towards induced pluripotency (Utikal et al., 2009). Another hypothesis is that *Tox4* is involved in the phosphorylation of RPB1, the catalytic subunit of RNA polymerase II (RNA Pol II), during release from RNA Pol II pausing via PP1 (Chen et al., 2008; Lee et al., 2010). Mechanistically, transcriptional pause release has been reported as a rate-limiting step during reprogramming to iPSCs (Liu et al., 2014a,b), where paused RNA Pol II assembles at the promoter of pluripotency genes during reprogramming, followed by pause release for productive transcription to take place (Fuda et al., 2009). Altogether, this would suggest that *Tox4* enables reprogramming via various mechanisms including mediating the proper closing and opening of chromatin nearby somatic and pluripotency genes, ensuring sufficient exogenous OKSM expression and by enabling timely cell cycle progression. We acknowledge that this model will need to be tested.

Furthermore, we identify *C2orf88* as a facilitator of reprogramming, and *Ube2a* as a barrier to reprogramming. Interestingly, a subset of these factors shows system-specific effects during reprogramming. For example, we found evidence that *Ube2a* acts as a barrier to reprogramming, consistent with a previous study (Cheloufi et al., 2015), but only in the presence of AA. These results may explain why a closely related family member, *Ube2i*, acts as a barrier to reprogramming in the presence (Cheloufi et al., 2015), but not in the absence of AA (Tahmasebi et al., 2014). Our result that *Bex2* knockdown has effects only in the absence of AA is in agreement with a recent study that proposed that high-efficiency reprogramming systems could compensate for the lack of *Bex2* during reprogramming (Schwarz et al., 2018). These results underscore the importance of comparing different reprogramming conditions, systems and stages for cell fate reprogramming studies.

Given that *Tox4* is involved in cell fate changes, it will be interesting to test whether this can be harnessed to direct cell fate and whether it contributes to diseases including cancer. TOX family genes have already been linked to epigenetic silencing in tumorigenesis (Tessema et al., 2012), proliferation and DNA damage repair in human T-cell acute lymphoblastic leukemia (Puch et al., 2011; Lobbardi et al., 2017). In addition, TOX family members are also involved in non-tumor diseases, such as pulmonary tuberculosis and HIV (Grant et al., 2013; Morchikh et al., 2013). One interesting outcome of our work is that *Tox4* may be relevant for the control of cell identity in regenerative medicine, human disorders and cancer therapy settings. To conclude, we identified *Tox4* as a novel transcriptional modulator of cell fate that mediates reprogramming from the somatic state to the pluripotent or neuronal fate. Mechanistically, TOX4 modulates proliferation and ensures sufficient ectopic TF expression, thereby allowing chromatin accessibility changes that are pivotal to reprogramming to take place early during reprogramming.

## MATERIALS AND METHODS

### Derivation of MEFs

MEFs were isolated at embryonic day (E)14.5 following removal of internal organs and head, followed by trypsin digestion and plating in MEF medium [DMEM (Gibco, 41966-052) supplemented with 10% (v/v) fetal bovine serum (FBS, Gibco, 10270-106), 1% (v/v) penicillin/streptomycin (P/S, Gibco, 15140-122), 1% (v/v) GlutaMAX (Gibco, 35050-061), 1% (v/v) non-essential amino acids (NEAA, Gibco, 11140-050) and 0.8% (v/v) β-mercaptoethanol (Sigma, M7522)]. For reprogramming experiments, MEFs derived from *Col1a1-tetO-OKSM* (Plath), *Rosa26-M2rtTA* mice were used (Sridharan et al., 2013). For transdifferentiation experiments to neurons, MEFs derived from *C57BL/6* mice were used. All animal work carried out in this study is covered by a project license approved by the KU Leuven Animal Ethics Committee.

### Cell culture and reprogramming

All cell lines used were tested for mycoplasma contamination at the start of each experiment. V6.5 ESCs were a gift from the laboratory of Dr Kathrin Plath (UCLA School of Medicine, USA). V6.5 ESCs were cultured on top of male WT feeders in mouse ESC medium [KnockOut DMEM (Gibco, 10829-018) supplemented with 15% FBS, 1% (v/v) P/S, 1% (v/v) GlutaMAX, 1% (v/v) NEAA, 0.8% (v/v) β-mercaptoethanol and mouse LIF]. X-GFP pre-iPSCs (Pasque et al., 2014) were grown in ESC medium on feeders and feeder-depleted a day before transfection. Pre-iPSCs were transfected with 3 µg transposase plasmid and 1 µg of either PB-NLS-Cherry or PB-Tox4 plasmid (see below). At 24 h after transfection, cells were selected with 20 µg/ml blasticidin for 48 h.

For reprogramming experiments, 15,000 MEFs were plated at passage 1–2 in each well of a 12-well plate precoated with gelatin (from porcine skin, 0.1% g/v final, Sigma, G2500) in mouse ESC medium (S/L condition). Reprogramming was induced by addition of 2 µg/ml DOX with or without the presence of 50 µg/ml AA for the next 12 to 15 days. Medium was replaced every 2 days. Alternatively, ESC medium was switched to KSR culture medium [where FBS is replaced by KSR (Gibco, 10828-028) in ESC media] on D4–D5 of reprogramming.

Reprogramming of pre-iPSCs was performed by switching pre-iPSCs to KSR medium in the presence of 2i/L [(GSK3 inhibitor CHIR-99021 (3 μM final, Axon Medchem, Axon 1386) and MEK inhibitor PD0325901 (1 μM final, Axon Medchem, Axon 1408)] with LIF.

Retroviral-mediated reprogramming was performed as described previously (Pasque et al., 2014). Briefly, MEFs at passages 1–3 were infected overnight at 50% confluency with pooled viral supernatant of individual pMX vectors encoding *Oct4*, *Sox2*, and *Klf4*, generated by transfecting PlatE, in ESC medium supplemented with 8 μg/ml polybrene (Sigma) and 50 µg/ml AA. A second round of retroviral infection was performed the next day. The following day, cells were split 1:5 onto irradiated feeder cells and 0.1% gelatin-coated plates in mESC medium supplemented with 50 µg/ml AA.

### RNAi

STEMCCA or Bl6 WT MEFs in 12-well plates were transfected with siRNA (20 nM final, Dharmacon) using 1.2 µl RNAi MAX (Invitrogen, 13778-150) for each well at D0 or/and every other day of reprogramming, as indicated in the figures. Knockdown efficiency was determined by RT-qPCR and western blotting. Information on individual siRNAs is listed in Table S11.

### AP staining

Cells were washed twice with PBS and stained for AP using the Vector Red Substrate kit (Vector, SK-5100) according to the manufacturer's instructions. Cells were then washed again with PBS and water, and colonies were counted after scanning the wells with a high-resolution scanner or Nikon eclipse Ti2 microscope.

### RT-qPCR

RT-qPCR was carried out largely as described previously (Song et al., 2019). Primer sequences are listed in Table S12. All assays used had an efficiency above 95%. Relative quantities of each transcript were calculated as arbitrary units from comparison to the standard curve. Relative expression level of the target transcript was presented as the ratio of the target transcript quantity to the housekeeping transcript quantity.

### Immunofluorescence

Immunofluorescence analyses were carried out largely as described previously (Pasque et al., 2014), using primary antibodies against the following proteins: NANOG (eBioscience, 14-5761 clone eBioMLC-51, 1:200; and Abcam, ab80892, 1:200), DPPA4 (R&D, AF3730, 1:200), TOX4 (Sigma, HPA017880, 1:100), EZH2 (BD, 612667, 1:200), TUJ1 (Covance, MMS-435P, 1:2000) and MAP2 (Synaptic Systems, 188002/6, 1:1000). Images were acquired using an ApoTome Zeiss Microscope equipped with an AxioCam MRc5 camera. For quantification, a colony was defined as positive when four or more closely localized or touching cells with clear nuclear staining for NANOG, DPPA4 or EZH2 were detected within a reprogramming culture, unless otherwise stated.

### Plasmid constructs

The full-length cDNAs of mouse *Tox4*, luciferase (from pGL2-Basic Promage, E1641), and NLS-Cherry were cloned into pENTR vectors (Invitrogen, K240020) with either a C-terminal or a N-terminal HA tag, or no tag, and recombined into pPB-CAG-Dest-pA-pgk-bSD (Addgene 74918) destination vectors. All constructs were verified by DNA Sanger sequencing.

### TOX4 overexpression in ESCs

ESCs (V6.5, grown on feeders in S/L conditions) were feeder-depleted before seeding in six-well plates pre-coated with 0.1% gelatin in S/L medium at a density of 650,000 cells per well, which were co-transfected with 1 µg of pPB expression constructs encoding Tox4 (HA-tagged or no tag) and 3 µg of pCAGP Base using 10 µl Lipofectamine 2000 (Invitrogen, 11668027). Transfected cells were selected with 20 µg/ml blasticidin (Fisher BioReagents, BP2647100) supplemented to the medium for 2 days starting from 24 h after transfection and maintained with 5 µg/ml blasticidin thereafter.

### Western blotting

Western blotting was carried out largely as described previously (Song et al., 2019), using the following primary antibodies: rabbit anti-TOX4 (Sigma, HPA017880, 1:1000; and Abcam, ab66651, 1:1000), mouse anti-ACTIN (Abcam, ab3280, 1:5000) and rabbit anti-GAPDH (Sigma, G9545, 1:1000) antibodies. Secondary antibodies were: HRP-conjugated goat anti-mouse-IgG antibody (Bio-Rad, 1706516, 1:5000) or goat anti-rabbit-IgG antibody (Bio-Rad, 1706515, 1:5000) for 30 min at room temperature. Data were analyzed with ImageJ.

### RNA-seq

Total RNA was isolated from *Tox4* and control siRNA-treated cells at D0 and D3 of reprogramming to induced pluripotency, MEFs and iPSCs using TRIzol following the manufacturer's protocol. Libraries were prepared as described before (Song et al., 2019). Libraries were pooled in equimolar amounts for single-end sequencing on an Illumina HiSeq 4000 instrument to yield ∼14.5 million (range 12–17 million) 51-bp-long reads per sample.

### Differential gene expression analysis

Reads were aligned to the mouse reference genome GRCm38/mm10 using STAR (v2.5.0a) with default parameters followed by conversion to BAM format sorted by coordinate. The mapping efficiency across samples was >79% of uniquely mapped reads. Next, the featureCounts function from the ‘Rsubread’ (v1.5.2) package in R (v3.5.2) was used to assign mapped reads to genomic features. The resulting read count matrix was used as input for the PCA, which included all variable genes. Differential gene expression analysis was performed using the DESEQ2 package (v1.21.22) in R (Love et al., 2014). A list containing all significantly differentially expressed genes (*P*<0.05) between *Tox4* siRNA and control siRNA-treated cells at D3 of reprogramming is provided in Table S13. *P*-values were corrected for multiple testing with the Benjamini–Hochberg method. Somatic genes were defined as the top 500 genes that were significantly more expressed in MEFs compared to iPSCs when sorting based on log2 fold change (adjusted *P*<0.05) in this dataset. Heatmaps were generated based on the unsupervised hierarchical clustering of both 500 most variable genes for the pluripotency-related and somatic-related gene lists using the pheatmap function in R.

### Enrichment analysis

Pathway enrichment and gene ontology (GO) analysis were performed using PANTHER on all significantly differentially expressed downregulated or upregulated genes between *Tox4* siRNA and control siRNA-treated cells at D3 of reprogramming (available in Tables S2–S5) with the following settings: analysis type, PANTHER overrepresentation test (released 20190606) (Thomas et al., 2003); annotation version and release date, GO ontology database released on 2019-02-02; reference list: *Mus musculus* all genes in database; and test type, Fisher's exact test with false discovery rate correction.

### CFSE staining, EdU staining and flow cytometry

Cells were pulse-labeled with the CellTrace™ CFSE Cell Proliferation Kit (Thermo Fisher Scientific, C34554) according to the manufacturer's instructions. Briefly, cells were incubated with 2 µM CFSE dissolved in PBS for 20 min at 37°C and washed twice with culture medium. For flow cytometry, cells were detached using 0.05% Trypsin-EDTA, resuspended at 10^5^ cells per 1 µl in 1× PBS with 0.5% BSA and 2 mM EDTA. Samples were stained with 1 µg/ml DAPI (Sigma, D9542) before analysis on a BD FACS Canto II HTS flow cytometer.

Non-synchronized cells were pulse-labeled with 10 μM EdU (Life Technologies) for 75 min. After detachment with 0.25% Trypsin-EDTA, cells were fixed with 4% PFA for 20 min, washed with PBS plus 2% FBS followed by 20 min permeabilization with PBS and 0.5% Triton X-100. After a PBS plus 2% FBS wash, cells were incubated with PBS containing 100 mM CuSO_4_, 1 M sodium ascorbate and 0.2 µM azide Alexa Fluor 647 for 10 min in the dark to reveal EdU incorporation. Samples were stained with 1 µg/ml DAPI before analysis on a BD FACS Canto II HTS flow cytometer. FlowJo was used as analysis software. The cycling index was calculated by calculating the proportion of cells in S and G_2_/M phase relative to cells in the G_0_ and G_1_ phase [(G_2_M+S)/(G_0_G_1_)].

### Omni-ATAC-seq

ATAC-seq was performed using the Omni-ATAC protocol as described previously (Corces et al., 2017; Song et al., 2019). Libraries were pooled in equimolar amounts for single-end sequencing on an Illumina HiSeq 4000 instrument to yield ∼28.75 million (range 22×10^6^–45×10^6^) 51 bp long reads per sample. Further processing resulted in 19 million (range 15×10^6^–28.5×10^6^) final reads on average with a minimal enrichment score of 10 at the transcriptional start site.

### Differential chromatin accessibility analysis

Single-end ATAC-seq raw data were analyzed using the ATAC-seq pipeline from the Kundaje laboratory (v1.1.5) with default parameters as described previously (Lee, 2016). Reads were aligned to the ENCODE mouse reference genome GRCm38/mm10 (ENCSR425FOI). Differential chromatin accessibility analysis was performed using the DiffBind (v2.10.0) package after which quantification occurred using the DESEQ2 (v1.21.22) and apeglm package (v1.4.2) in R (Love et al., 2014; Zhu et al., 2019). *P*-values were corrected for multiple testing with the Benjamini–Hochberg method. Pluripotency accessible regions were defined as the top 500 regions significantly more open in iPSCs compared to MEFs when sorting based on log2 fold change (adjusted *P*<0.05) in this dataset. Somatic accessible regions were defined as the top 500 regions significantly more open in D0 MEFs compared to iPSCs when sorting based on log2 fold change (adjusted *P*<0.05) in this dataset. Heatmaps were generated by using the pheatmap function (v1.0.10) in R. Boxplots were generated using the ggplot2 (v3.0.0) package in R. The function of cis-regulatory regions was predicted using GREAT (v3.0.0) using mouse NCBI build 38 (UCSC mm10, Dec/2011) as species assembly with gene regulatory domain function defined as the single nearest gene within 1000 kb (McLean et al., 2010).

### Neuronal transdifferentiation

25,000 MEFs were plated at early passage in each well of a 12-well plate precoated with 1:30 DMEM/F12 diluted hESC qualified Matrigel (Corning, 354277) in MEF medium. MEFs were transduced with FUW-TetO-*Ascl1* (Addgene 27150), FUW-TetO-*Myt1l* (Addgene 27152), FUW-TetO-*Brn2* (Addgene 27151) and FUW-M2rtTA (Addgene 20342) lentiviruses (Hockemeyer et al., 2008; Vierbuchen et al., 2010). Transdifferentiation was induced the next day by the addition of 2 µg/ml DOX over the next 14 days. At 2 days after infection, the medium was changed to N3 medium [DMEM-F12, 25 μg/ml insulin (Sigma), 50 µg/ml transferrin (Sigma), 30 nM sodium selenite (Sigma), 20 nM progesterone (Sigma), 100 nM putrescine (Sigma), 10 ng/ml FGF2 (R&D Systems, Wiesbaden-Nordenstadt, Germany), penicillin/streptomycin and 1× Glutamax] supplemented with 2 µg/ml DOX for the remainder of the experiment. *Tox4* siRNA3 and control siRNA2 were individually transfected into early passage WT male Bl6 MEFs every other day throughout the transdifferentiation using Lipofectamine-RNAi MAX following the manufacturer's recommendations. Medium was replaced every day. At D14, cells were fixed using 4% PFA as described previously (Song et al., 2019).

### Statistical analysis

Statistical tests were performed using the stats package (v3.5.2) in R, GraphPad Prism 7 (GraphPad Software) and Excel. Wilcoxon rank sum test with FDR correction, one-way ANOVA with Dunnett's multiple comparisons test and Student's *t*-test were used as indicated. All data, unless indicated otherwise, are presented as the mean±s.d. *P* values of <0.05 were considered statistically significant.

## Supplementary Material

Supplementary information
