# Supplementary material for: Tox4 modulates cell fate reprogramming
Source: J Cell Sci. 2019 Oct 22;132(20):jcs232223. doi: 10.1242/jcs.232223 (PMC6826012; doi:10.1242/jcs.232223)
Supplement: Supplementary information [file joces-132-232223-s1.pdf]

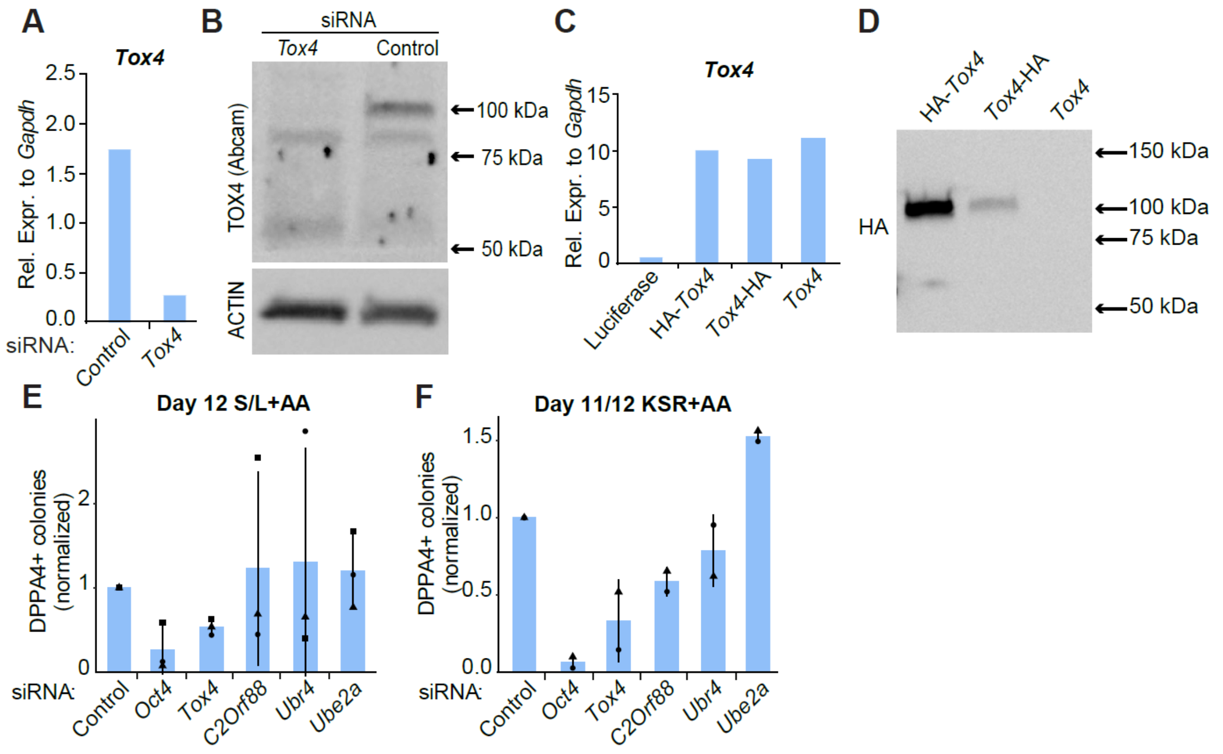

Figure S1. Validation of *Tox4* siRNA knockdown in primary screen.

(A) *Tox4* transcript level after 4 days of STEMCCA MEFs reprogramming and transfection of *Tox4* or control siRNAs every 2 days. Results are shown relative to the expression of *Gapdh* (arbitrary units). Results are shown as the mean of technical duplicates, (n=1).

(B) Western blot analysis for TOX4 (Abcam antibody) and ACTIN after 4 days of STEMCCA MEFs reprogramming and transfection of *Tox4* or control siRNAs every other day. Results are shown for the same experiment as in (A), (n=1).

(C) *Tox4* transcript level in ESCs with ectopic expression of Luciferase, HA-*Tox4*, *Tox4*-HA or untagged *Tox4*. Results are shown relative to the expression of *Gapdh* (arbitrary units), (n=1).

(D) Western blot analysis for HA in ESCs with ectopic expression of HA-*Tox4*, *Tox4*-HA or untagged *Tox4*, (n=1).

(E) The number of DPPA4 colonies at D12 of reprogramming in S/L+AA. Counts were normalized to counts in control conditions. Results are shown as the normalized mean +/- s.d., (n=3 with biological duplicates in total). 1-way ANOVA with Dunnett's multiple comparisons test compared to control.

(F) The number of DPPA4 colonies at D11/12 of reprogramming in KSR+AA. Counts were normalized to counts in control conditions. Results are shown as the normalized mean +/- s.d. (n=2 with biological duplicates in total). 1-way ANOVA with Dunnett's multiple comparisons test compared to control. Squares, triangles and circles represent one independent experiment each.

Journal of Cell Science • Supplementary information

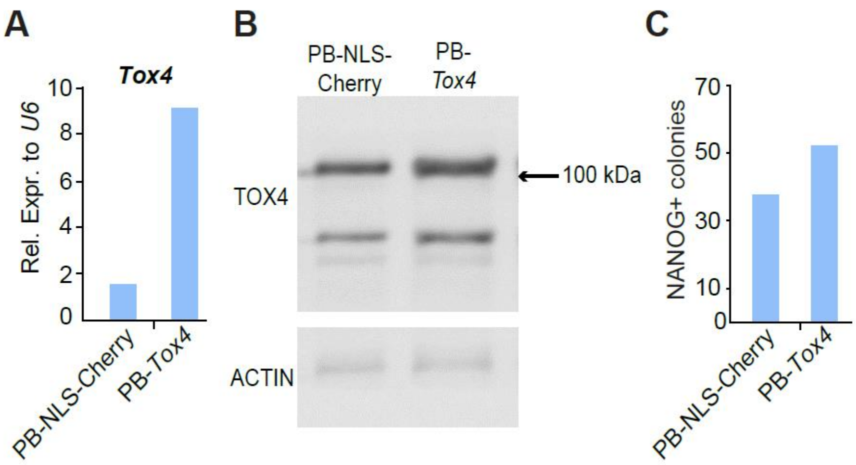

Figure S2. *Tox4* overexpression does not affect reprogramming of pre-iPSCs towards pluripotency.

(A) *Tox4* transcript level after 9 days of *Tox4* or control NLS-cherry overexpression in pre-iPSCs. Results are shown as the mean of technical duplicates, relative to the expression of U6 (arbitrary units) (n=1).

(B) Western blot for TOX4 (Sigma) and ACTIN after 9 days of reprogramming pre-iPSCs while overexpressing Tox4 or NLS-cherry, (n=1).

(C) The number of NANOG+ colonies after 9 days of reprogramming pre-iPSCs while overexpressing TOX4 of NLS-cherry. Results are shown as the mean of two technical duplicates, (n=1).

Journal of Cell Science • Supplementary information

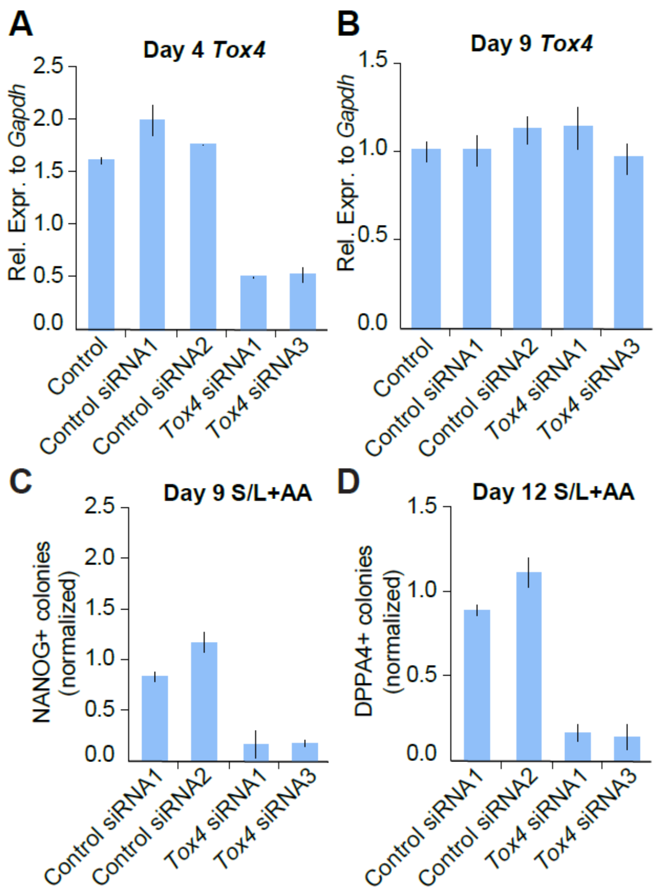

Figure S3. *Tox4* knockdown using individual siRNA decreases induced pluripotency reprogramming efficiency.

(A) *Tox4* transcript level after 4 days of STEMCCA MEFs reprogramming and transfection of *Tox4* or control siRNAs on D0 and D2. Results are shown relative to the expression of *Gapdh* (arbitrary units). Results are shown as the mean of biological duplicates +/- s.d. (n=1).

(B) *Tox4* transcript level after 9 days of STEMCCA MEFs reprogramming and transfection of *Tox4* or control siRNAs on D0 and D2. Results are shown relative to the expression of *Gapdh* (arbitrary units). Results are shown as the means of biological duplicates +/- s.d. (n=1).

(C) The number of NANOG+ colonies at D9 of reprogramming in S/L+AA. Counts were normalized to counts in control conditions. Results are shown as the normalized mean of biological duplicates +/- s.d. (n=1).

(D) The number of DPPA4+ colonies at D12 of reprogramming in S/L+AA. Counts were normalized to counts in control conditions. Results are shown as the normalized mean of biological duplicates +/- s.d. (n=1).

Journal of Cell Science • Supplementary information

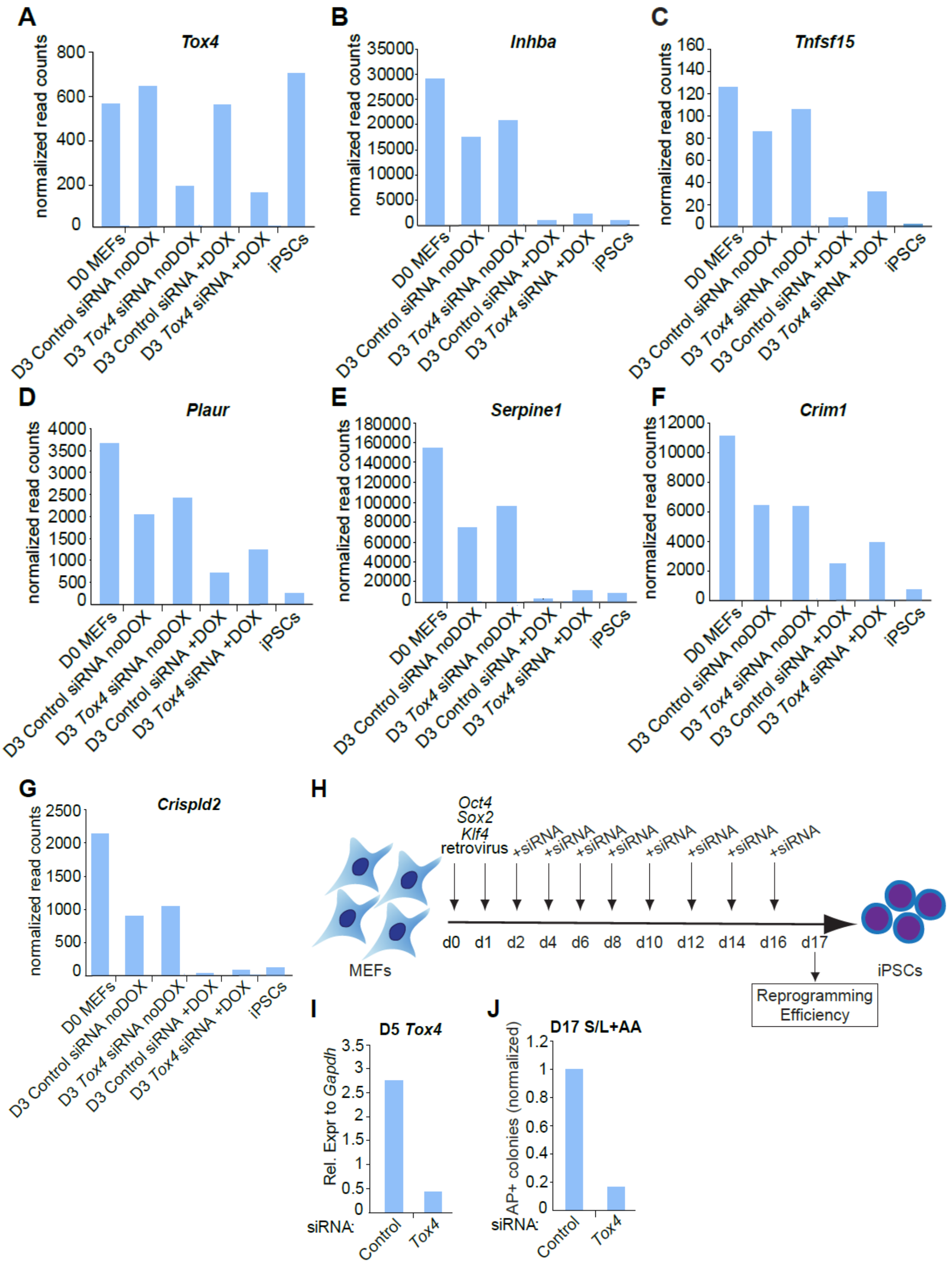

Figure S4. Transcriptional changes throughout reprogramming to induced pluripotency upon *Tox4* depletion.

(A) *Tox4* transcript level at different time points of STEMCCA MEFs reprogramming after transfection of *Tox4* or control siRNAs on D0 and D2. Results are shown as the mean normalized read counts of technical duplicates, (n=1).

Journal of Cell Science • Supplementary information

(B-M) *Inhba* (B), *Tnfsf5* (C), *Plaur* (D), *Serpine1* (E), *Crim1* (F), *Crispld2* (G) transcript level at different time points of STEMCCA MEFs reprogramming after transfection of *Tox4* or control siRNAs on D0 and D2. Results are shown as the mean normalized read counts of technical duplicates, (n=1).
(H) Schematic of retroviral mediated reprogramming of Bl6 WT MEFs in S/L +AA combined with a siRNA-mediated somatic *Tox4* knockdown every two days throughout reprogramming.
(I) *Tox4* transcript level at D5 of retroviral mediated reprogramming after transfection of *Tox4* or control siRNAs on D2 and D4. Results are shown as the mean of technical duplicates relative to the expression of *Gapdh* (arbitrary units), (n=1).
(J) The number of AP+ colonies at D17 of reprogramming in S/L +AA. Colony counts were normalized to colony counts in control conditions. Results are shown as the mean of technical duplicates, (n=1).

Journal of Cell Science • Supplementary information

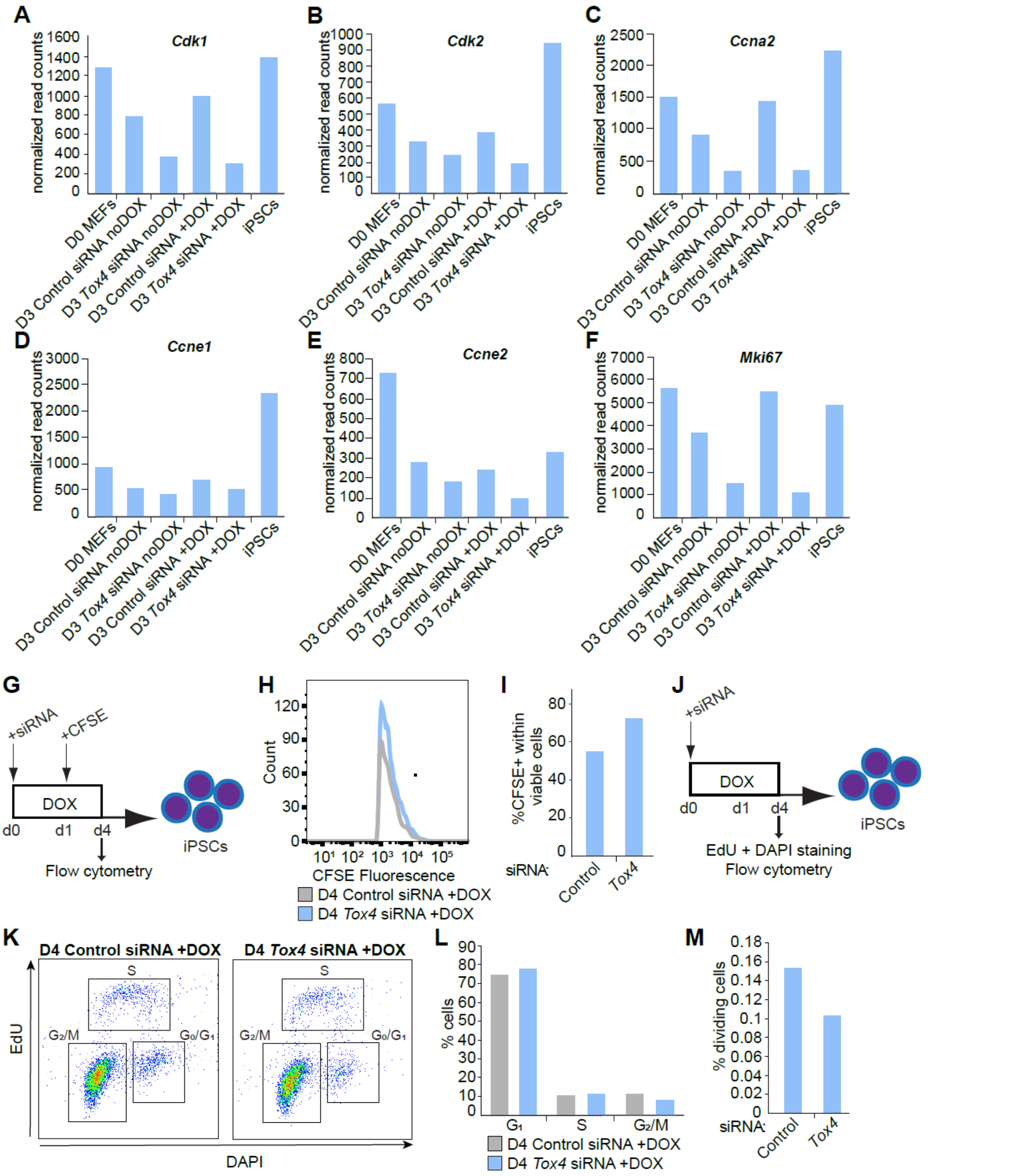

Fig. S5. Cell proliferation decreased throughout reprogramming to induced pluripotency upon *Tox4* depletion.

(A-F) *Cdk1* (A), *Cdk2* (B), *Ccna2* (C), *Ccne2* (D), *Ccne1* (E) and *MKi67* (F) transcript level at different time points of STEMCCA MEFs reprogramming after transfection of *Tox4* or control siRNAs on D0 and D2. Results are shown as the mean normalized read counts of technical duplicates, (n=1).

(G) Schematic of siRNA-mediated somatic *Tox4* knockdown at the start of reprogramming to iPSCs. *Tox4* was targeted at D0 by siRNA transfection of STEMCCA MEFs after subsequent DOX induction

Journal of Cell Science • Supplementary information

of reprogramming. Cells were labeled with CFSE at D1 and analyzed by flow cytometry at D4 of reprogramming.

(H) Histogram representing the flow cytometry analysis of the proportion of CFSE+ cells within the viable (DAPI negative) cell population at D4 of reprogramming for *Tox4* siRNA (blue) and control condition (grey).

(I) Quantification of histograms represented in (H). Results are shown as the mean of technical duplicates, (n=1).

(J) Schematic of siRNA-mediated somatic *Tox4* knockdown at the start of reprogramming to iPSCs. *Tox4* was targeted at D0 by siRNA transfection of STEMCCA MEFs after subsequent DOX induction of reprogramming. Cells were stained with EdU and analyzed by flow cytometry at D4 of reprogramming. Results are shown as the mean of technical duplicates, (n=1).

(K) Density plot representing the EdU and DAPI flow cytometry analysis of the viable (DAPI negative) cell population at D4 of reprogramming, with 6451 Control siRNA and 5721 *Tox4* siRNA treated cells, respectively.

(L) Quantification of gated population representing G_0/M, S and G_0/G_1 in (K) (n=1).

(M) The proportion of dividing cells, defined as the proportion of cells in S and G_2/M phase relative to cells in the G_0/G_1 phase (S+G_2M) / G_0G_1 of *Tox4* and control siRNA treated cells at D4 of reprogramming. Results are shown as the mean of technical duplicates, (n=1).

Journal of Cell Science • Supplementary information

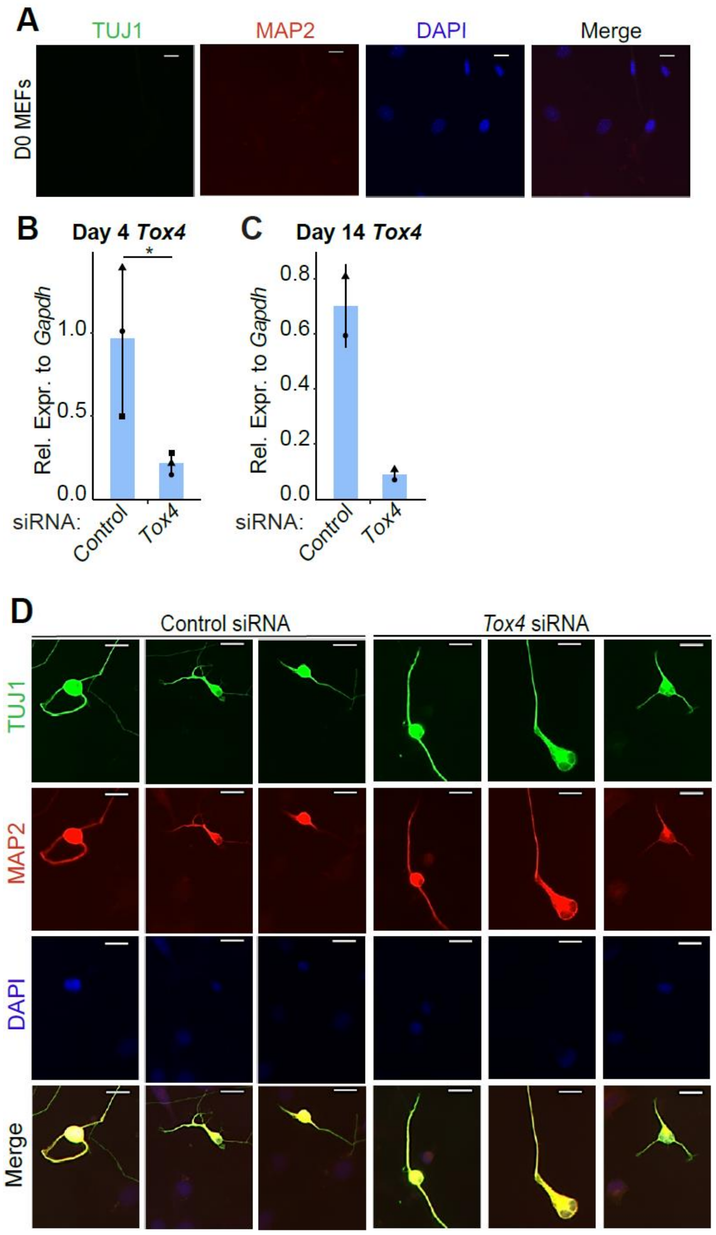

Fig. S6. Fibroblasts are TUJ1 and MAP2 negative before transdifferentiation to iNs.

(A) Immunofluorescence analysis for TUJ1/MAP2 at D0 of transdifferentiation. Representative images examined for TUJ1 (green), MAP2 (red) and DAPI (blue, nuclei counterstaining) are shown. Scale bar, 20 µm.

(B-C) *Tox4* transcript level at D4 (B) and D14 (C) of transdifferentiation and transfection of *Tox4* or control siRNAs every 2 days. Results are shown as the normalized mean relative to the expression of *Gapdh* (arbitrary units) +/- s.d. (n=3/2 respectively with 1 biological replicate in total). Two-tailed unpaired t test, * $p<0.05$. Squares, triangles and circles represent one independent experiment each.

(D) Immunofluorescence analysis for TUJ1/MAP2 at D14 of transdifferentiation for control and *Tox4* siRNA condition. Representative images examined for TUJ1 (green), MAP2 (red) and DAPI (blue, nuclei counterstaining) are shown. Scale bar, 20 µm.

Table S1. Overview of somatic genes defined based on RNA-seq data.
Somatic genes were defined as the top 500 genes which were significantly more expressed in MEFs compared to iPSCs when sorting based on log2 Fold Change (p adjusted <0.05). The table below includes names, log2 Fold Change and adjusted p value of somatic genes. This table is related to Fig. 4.

Click here to Download Table S1

Table S2. Go Slim Molecular Function Gene Ontology terms associated with significantly differentially expressed genes which are upregulated in NTC siRNA treated cells at D3 of reprogramming.
Overview of Go Slim Molecular Function Gene Ontology terms associated with all significantly differentially expressed genes which are upregulated in NTC siRNA treated cells at D3 of reprogramming (p adjusted <0.05). The table below includes Gene Ontology terms, Fold Enrichment and False Discovery Rate.

Click here to Download Table S2

Table S3. Go Slim Biological Processes Gene Ontology terms associated with significantly differentially expressed genes which are upregulated in NTC siRNA treated cells at D3 of reprogramming.
Overview of Go Slim Biological Processes Gene Ontology terms associated with all significantly differentially expressed genes which are upregulated in NTC siRNA treated cells at D3 of reprogramming (p adjusted <0.05). The table below includes Gene Ontology terms, Fold Enrichment and False Discovery Rate.

Click here to Download Table S3

Journal of Cell Science • Supplementary information

Table S4. Go Slim Molecular Function Gene Ontology terms associated with significantly differentially expressed genes which are upregulated in *Tox4* siRNA treated cells at D3 of reprogramming.

Overview of Go Slim Molecular Function Gene Ontology terms associated with all significantly differentially expressed genes which are upregulated in *Tox4* siRNA treated cells at D3 of reprogramming (p adjusted <0.05). The table below includes Gene Ontology terms, Fold Enrichment and False Discovery Rate.

Click here to Download Table S4

Table S5. Go Slim Biological Processes Ontology terms associated with significantly differentially expressed genes which are upregulated in *Tox4* siRNA treated cells at D3 of reprogramming.

Overview of Go Slim Biological Processes Gene Ontology terms associated with all significantly differentially expressed genes which are upregulated in *Tox4* siRNA treated cells at D3 of reprogramming (p adjusted <0.05). The table below includes Gene Ontology terms, Fold Enrichment and False Discovery Rate.

Click here to Download Table S5

Table S6. Overview of somatic chromatin regions defined based on ATAC-seq data.

Somatic accessible regions were defined as the top 500 regions significantly more open in D0 MEFs compared to iPSCs when sorting based on log2 Fold Change (p adjusted <0.05). The table below includes peak identifiers, log2 Fold Change and adjusted p value of somatic chromatin regions. This table is related to Fig. 5D.

Click here to Download Table S6

Journal of Cell Science • Supplementary information

Table S7. List of somatic chromatin regions that closed with a delay in *Tox4* siRNA-treated cells compared to control conditions.

Somatic chromatin regions that closed with a delay were defined based on visual inspection of Figure 5D. The table below includes peak identifiers, chromosome and genomic starting and ending location of somatic chromatin regions.

Click here to Download Table S7

Table S8. Overview of pluripotency chromatin regions defined based on ATAC-seq data.

Pluripotency accessible regions were defined as the top 500 regions significantly more open in iPSCs compared to MEFs when sorting based on log2 Fold Change (p adjusted <0.05). The table below includes peak identifiers, log2 Fold Change and adjusted p value of pluripotency chromatin regions. This table is related to Fig. 5F.

Click here to Download Table S8

Table S9. List of pluripotency chromatin regions that opened with a delay in *Tox4* siRNA-treated cells compared to control conditions.

Pluripotency chromatin regions that opened with a delay were defined based on visual inspection of Fig. 5F. The table below includes peak identifiers, chromosome and genomic starting and ending location of somatic chromatin regions.

Click here to Download Table S9

Table S10. Compiled list of genes which were associated with pluripotency chromatin regions that opened with a delay in Tox4 siRNA-treated cells compared to control conditions.

Gene association was performed using GREAT with regions described in Table S9 used as input.

Click here to Download Table S10

Table S11. Overview of siRNA used in this study.

The table below includes the siRNA name used in this study, official siRNA name, company and catalog number.

siRNA name	Product description	Company	Catelog number
Control siRNA	ON-TARGETplus Non-targeting Pool	Dharmacon	D-001810-10-05
Tox4 siRNA	Tox4 SMARTpool ON-TARGETplus siRNA	Dharmacon	L-044493-01-0005
Tox4 siRNA	Set of 4 Upgrade: ON-TARGETplus Tox4 siRNA	Dharmacon	LU-044493-01-0005
Control siRNA2	ON-TARGETplus Non-targeting siRNA #2	Dharmacon	D-001810-02-05
Oct4 siRNA	Pou5f1 SMARTpool ON-targetPLus siRNA	Dharmacon	L-046256-00-0005
Chaf1a siRNA	Chaf1a SMARTpool ON-TARGETplus siRNA	Dharmacon	L-060606-00-0005
Bex2 siRNA	Bex2 SMARTpool ON-TARGETplus siRNA	Dharmacon	L-043921-01-0005
C2Orf88 siRNA	C2orf88 SMARTpool ON-TARGETplus siRNA	Dharmacon	L-053340-01-0005
Tcl1a siRNA	Tcl1a SMARTpool ON-TARGETplus siRNA	Dharmacon	L-062391-01-0005
Bcor siRNA	Bcor SMARTpool ON-TARGETplus siRNA	Dharmacon	L-058762-01-0005
Ubr4 siRNA	Ubr4 SMARTpool ON-TARGETplus siRNA	Dharmacon	L-050850-00-0005
Zhx siRNA	Zhx3 SMARTpool ON-TARGETplus siRNA	Dharmacon	L-059734-01-0005
Ube2a siRNA	Ube2a SMARTpool ON-TARGETplus siRNA	Dharmacon	L-061675-00-0005
Alkbh1 siRNA	Alkbh1 SMARTpool ON-TARGETplus siRNA	Dharmacon	L-043852-00-0005

Table S12. Primer sequences.

Click here to Download Table S12

Table S13: Overview of all significantly differentially expressed genes between *Tox4* siRNA and NTC siRNA treated cells at D3 of reprogramming.

All significantly differentially expressed genes between *Tox4* siRNA and NTC siRNA treated cells at D3 of reprogramming were generated based on DESEQ2 analysis of RNA-seq data (p adjusted <0.05). The table below includes names, log2 Fold Change and adjusted p value of differentially expressed genes. This table is related to Figure 4.

Click here to Download Table S13

Journal of Cell Science • Supplementary information
